# Interpreter and limited-English proficiency patient training helps develop medical and physician assistant students’ cross-cultural communication skills

**DOI:** 10.1186/s12909-024-05173-z

**Published:** 2024-02-23

**Authors:** Quennie Nguyen, Julia Flora, Preetha Basaviah, Madika Bryant, Poonam Hosamani, Jerri Westphal, John Kugler, Jason Hom, Jeffrey Chi, Johanna Parker, Alicia DiGiammarino

**Affiliations:** 1 Stanford University, Stanford, CA 94305 USA; 2 Department of Primary Care and Population Health, Stanford University School of Medicine, Stanford, CA 94305 USA; 3 Stanford University School of Medicine, 1265 Welch Rd, Stanford, CA 94305 USA; 4 Division of Hospital Medicine, Stanford University School of Medicine, Stanford, CA 94305 USA; 5 Stanford Health Care, Stanford, CA 94305 USA

**Keywords:** Health equity, Communication, Interpretation

## Abstract

**Background:**

The increasing linguistic and cultural diversity in the United States underscores the necessity of enhancing healthcare professionals' cross-cultural communication skills. This study focuses on incorporating interpreter and limited-English proficiency (LEP) patient training into the medical and physician assistant student curriculum. This aims to improve equitable care provision, addressing the vulnerability of LEP patients to healthcare disparities, including errors and reduced access. Though training is recognized as crucial, opportunities in medical curricula remain limited.

**Methods:**

To bridge this gap, a novel initiative was introduced in a medical school, involving second-year students in clinical sessions with actual LEP patients and interpreters. These sessions featured interpreter input, patient interactions, and feedback from interpreters and clinical preceptors. A survey assessed the perspectives of students, preceptors, and interpreters.

**Results:**

Outcomes revealed positive reception of interpreter and LEP patient integration. Students gained confidence in working with interpreters and valued interpreter feedback. Preceptors recognized the sessions' value in preparing students for future clinical interactions.

**Conclusions:**

This study underscores the importance of involving experienced interpreters in training students for real-world interactions with LEP patients. Early interpreter training enhances students' communication skills and ability to serve linguistically diverse populations. Further exploration could expand languages and interpretation modes and assess long-term effects on students' clinical performance. By effectively training future healthcare professionals to navigate language barriers and cultural diversity, this research contributes to equitable patient care in diverse communities.

**Supplementary Information:**

The online version contains supplementary material available at 10.1186/s12909-024-05173-z.

## Background

The influx of immigrants and growing racial minority populations has amplified the diversity within the United States (US), leading to a greater variety of languages. The 2022 American Community Survey Report highlighted that approximately 69.2 million, or just over 1 in 5, Americans aged five or older speak a non-English language at home [1]. Of those who speak another language, 38.3% or 26.5 million individuals, speak English “less than very well”. The US Department of Justice uses the acronym “LEP”, or “limited-English proficient”, to refer to individuals whose primary spoken language is not English and who may possess limited proficiency in reading, writing, speaking, and/or understanding English [2]. There is an increasing need for the healthcare system to strengthen resources and individual clinician skills to accommodate this linguistically diverse population.

LEP patients are particularly vulnerable to healthcare disparities [3]. Studies have shown that lack of comprehension of healthcare information can lead to inadequate understanding before providing medical consent, higher risk of medical errors [4, 5], decreased medical adherence [6], and gaps in health insurance coverage [7]. For Hispanic and Asian-American LEP patients, language barriers are a significant challenge to accessing preventative services or healthcare at all, especially among older individuals [8, 9]. Providing trained medical interpreters to LEP patients has been shown to significantly improve patient satisfaction [10, 11]. Title VI of the 1964 Civil Rights Act mandated that healthcare providers provide access to professional language services free of cost to LEP patients [12]. The 2013 Enhanced National Standards for Culturally and Linguistically Appropriate Services (CLAS) in Health Care reinforce this mandate with guidelines for healthcare organizations to provide language assistance to LEP patients, inform individuals of availability of these services, and ensure competency of individuals providing language assistance. Healthcare providers who regularly work with LEP populations have also expressed the desire for additional training for working with medical interpreters. These factors demonstrate the necessity for early and collaborative training of healthcare students with certified medical interpreters.

Despite this need, opportunities to work with LEP patients in U.S. medical curricula are underdeveloped. A 2018 survey of 147 Liaison Committee on Medical Education (LCME) accredited U.S. medical schools asked if students are provided with formal instruction on working with medical interpreters and/or LEP patients [13]. Thirty-eight schools (26%) responded to the survey, and, of these schools, the majority did offer some sort of curriculum to prepare students to work with medical interpreters and/or LEP patients. These programs included scripted interactions with standardized LEP patients and didactic lecture sessions. Though interviewing standardized LEP patients is valuable for developing students’ cross-linguistic communication skills, contact with real hospital patients and certified medical interpreters can increase the instructive authenticity of the experience. This study aims to assess a new educational initiative at our medical school: to complement first-year training with standardized LEP patients, second-year medical and physician assistant (PA) students worked with real LEP patients and received instruction from medical interpreters in clinical practicum sessions.

## Methods

### Research context

The Practice of Medicine (POM) course at our medical school provides clinical preparation for first- and second-year medical and PA students [14]. Participation in this course provides students with a foundation in health policy, medical ethics, nutrition, clinical epidemiology, behavioral medicine, nutrition, population health, information literacy, and quantitative medicine. Additionally, students learn the hands-on basics of the medical interview, physical examination, and clinical reasoning. During the clinical reasoning component, students develop skills in gathering, organizing, synthesizing, interpreting, and communicating clinical information through interactive sessions that integrate closely with the basic science and pathophysiology courses.

During the first year of POM, students receive training in best practices for working with an interpreter. They then practice these skills during an encounter with a standardized patient and receive feedback from an observing faculty member. In second year, students engage in a two-quarter clinical practicum experience during which they receive training and mentorship at a hospital site. During these sessions, students conduct a patient encounter with a previously consented patient, give an oral presentation on the patient case, and complete a formal, written History and Physical or Subjective, Objective, Assessment, and Plan (SOAP) note. Experienced clinical preceptors then provide constructive verbal and written feedback on the students’ performance on each component of the session. The clinical practicum is also designed to allow the student to develop a relationship with a preceptor who serves as a role model, mentor, and educator.

As part of our new educational initiative, second year students received a brief didactic refresher on skills for working with an interpreter. Each practicum group of three students and a faculty preceptor then had one session dedicated to working with a professional Spanish medical interpreter and one Spanish-speaking LEP patient. As the second most-spoken language in the US other than English, Spanish is spoken in 74% of LEP patient-provider encounters nationally and was chosen as the language of focus for this clinical program [15]. Patients at our institution provide their language preference (for both spoken and written communication) at the time of admission, information that is then featured prominently in the medical record system. Additionally, when needed, signage is posted in patient rooms that states language preference and contact information for interpreter services. Before the patient encounter, the interpreter provided guidance on best practices for working with an interpreter and offered an opportunity for the students to ask questions. Students then worked with the interpreter to interview and physically examine a Spanish-speaking patient, who was consented prior to the practicum session. After the session, the interpreter provided constructive feedback directly to the students.

### Data collection

Following completion of the clinical practicum sessions, a retrospective analysis survey was designed in Qualtrics and distributed via email to 43 medical and PA students, 15 preceptors, and 6 interpreters for a total of 64 possible respondents. Additional files 1, 2, and 3 show the surveys sent to students, preceptors, and interpreters respectively. The survey consisted of both open-ended and 5-point Likert scale questions serving to assess understanding of session expectations, perceived benefit of interpreter recommendations and feedback, and overall perceived value of integrating LEP patient experiences and interpreter instruction into medical education. All survey responses were anonymous.

### Data analysis

Numerical analysis of respondent agreement with statement prompts was applied to the Likert scale, with scores 4 (“agree”) and 5 (“strongly agree”) being combined to reflect an overall percentage of agreement. All responses were analyzed by descriptive statistics using both the Qualtrics software and Microsoft Excel (2021), with graphs being generated in GraphPad Prism 10 (2023). Responses to the open-ended questions were read to group and identify common themes among the three surveyed populations (preceptors, students, and interpreters).

The data collection protocol was exempted by the Stanford Institutional Review Board. Reporting followed SQUIRE reporting guidelines for quality improvement studies.

## Results

Of the 64 respondents who received the survey, 32 individuals responded (16 students, 11 preceptors, and 5 interpreters), reflecting a 37.2%, 73.3%, and 83.3% response rate for students, preceptors, and interpreters, respectively. All responses were analyzed.

### Perspectives on preparation and discussion before patient encounters

**Fig. 1:**
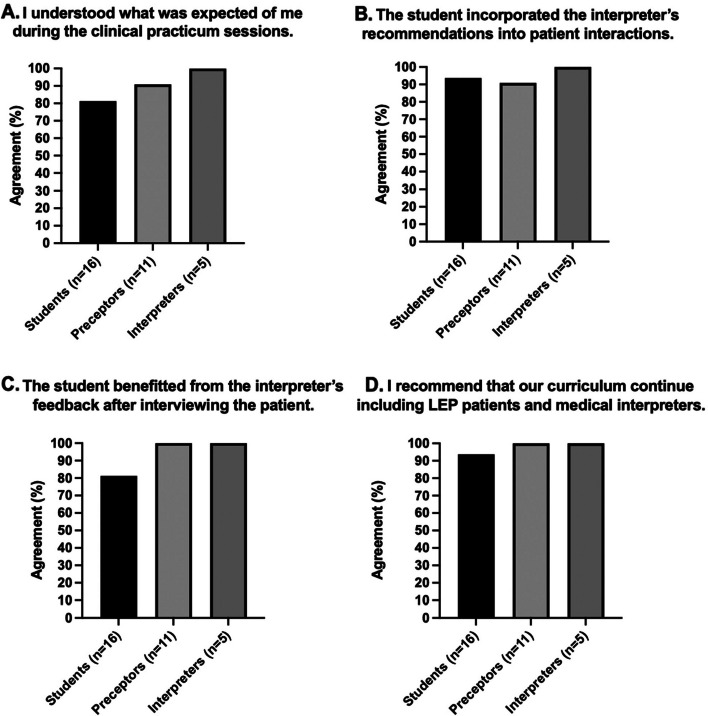
Medical and PA students, preceptors, and interpreters alike believe that inclusion of interpreters and LEP patients in medical training is valuable. The figures display Likert scale responses to prompts regarding (**A**) preparedness for the clinical practicum sessions, (**B**) the incorporation of interpreter recommendations prior the patient interview. (**C**) The benefit of interpreter feedback after the patient interview. (**D**) If the curricular intervention should be continued. Percent agreement includes 4 (“agree”) and 5 (“strongly agree”) scale rates

In response to the Likert scale questions, most respondents (87.5%, *n* = 28/32) understood what was expected of them during the practicum sessions (Fig. 1A). 90.9% (*n* = 10/11) of preceptors noted that the interpreter provided recommendations to the students prior to seeing the patient, with 93.8% (*n* = 30/32) of all respondents indicating that such recommendations were incorporated into the students’ interviews (Fig. 1B). Most preceptors (90.9%, *n* = 10/11) also agreed that the students benefited from the instruction provided by the interpreter prior to seeing the patient, and all interpreters (100.0%, *n* = 5/5) stated that the preceptor invited their input in the discussion before the patient interview.

In the open-ended questions, all respondents were asked how the set-up and preparation of the experience could be improved for future sessions. Preceptors and interpreters commonly mentioned the time constraint involved in including medical interpreters during the patient interview. One preceptor suggested that both students and preceptors could be provided a guide ahead of time about best practices in working and teaching with interpreters. An interpreter similarly suggested having more time specifically allocated for providing students with “background information” on their role and training as professional medical interpreters. Overall, however, most respondents commended the program for robustly preparing students for LEP patient interviews.

### Reflection on feedback and session value after patient encounters

Most students (81.3%, *n* = 13/16) felt that they benefited from the interpreter’s feedback after each session (Fig. 1C). The same proportion also felt more confident working with an interpreter after the experience. In their written responses, a common theme among students was the appreciation of practical experience “rather than a theoretical discussion.” They noted that “the experience is quite different from the controlled standardized patient environment,” and identified certain techniques that helped them work with LEP patients and interpreters, such as focusing on “using concise language,” making eye contact, and speaking directly to the patient.

All preceptors (100.0%, *n* = 11/11) noted that the clinical practicum session with real LEP patients and interpreters prepares students for similar interactions as clinicians. Preceptors wrote in response to the open-ended survey questions that continued training with interpreters can also be practical among current health care professionals; many themselves appreciated learning how to logistically access interpreter services at Stanford Hospital. They commonly used words like “valuable” and “practical on-the-ground experience” to emphasize the real-life opportunity to practice in a defined setting. Notably, preceptors also connected the impact of interpreter collaboration to larger social and healthcare ideas, citing how the program develops “cultural awareness” and “an appreciation for the diversity of our patient population”.

Ultimately, the practicum sessions were well-received by participants, with 96.9% (*n* = 31/32) of all respondents recommending that LEP patients and medical interpreters continue to be integrated into the pre-clerkship curriculum (Fig. 1D). When asked what they appreciated about the experience, interpreters found it valuable to introduce their roles and what their “job entails” to clinicians in training to “create awareness” of the importance of interpreter services’ engagement with LEP patients.

## Discussion

Students, preceptors, and interpreters agreed that the clinical sessions with interpreters and LEP patients proved to be a valuable curricular intervention to prepare students for future interactions as clinicians. Most respondents, regardless of role, felt prepared for the clinical preparation sessions. Interpreters were able to provide both pre-interview recommendations to students as well as post-session feedback, which students and preceptors acknowledged as beneficial. Students felt more confident working with medical interpreters and LEP patients after the clinical sessions.

Our data suggests that integrating interpreters into pre-clerkship clinical experiences may strengthen communication with linguistically diverse populations. Students benefit when interpreters are actively involved in their training by answering questions, providing recommendations and feedback, and fostering a collaborative learning environment. This work is significant because it is the first time that many of our medical students experience working with an interpreter. Some of our students do have the opportunity to work with interpreters as a part of their required shifts at the Cardinal Free Clinics, student-run primary and specialty care clinics that treat underserved patients in San Jose, CA and Redwood City, CA. In contrast to first-year training, where there are actors who are trained to embody the role of medical interpreters, inclusion of experienced medical interpreters as key pedagogical figures in medical and PA students’ training allows students to navigate the dynamic and dialogue with real interpreters. Furthermore, clinical preceptors have the opportunity to reinforce their own skills working with interpreters. Early-career resident physicians have been shown to underuse interpreter services even when they are available [16], but a survey of residents has also shown that those who received cross-cultural training feel more confident caring for a diverse patient population [17].

This study provides valuable data that builds upon recent attention focused upon the role of early training and medical education in improving care for diverse patient populations [18]. In the age of development of cross-cultural diversity curricula in both pre-medical and medical education [19, 20], as well as narrative medicine that acknowledges the relevance of sociocultural contexts along with patients’ physical symptoms [21], progress is being made to prepare students to care for patients from diverse backgrounds. Accrediting bodies such as the LCME and the Accreditation Council for Graduate Medical Education have also updated cultural humility requirements for medical training, specifically the acknowledgement of language barriers as a root cause of health disparities and skillful training of interpreter services to address these barriers [3, 22, 23]. Existing literature has noticed the lack of strategies to evaluate the impact of these curricular intervention [18], so surveys like the one conducted in this study provide one framework for evaluation that assess attitudes of students, preceptors, and interpreters.

For future studies, this pilot program could be expanded to include more languages and telephone (or video) interpreters. For the pilot year of the second-year clinical practicum sessions, only Spanish-speaking LEP patients were included due to logistical constraints. In-person interpretation has the added benefit of making use of non-verbal cues and managing the flow of communication in complex situations. However, there are often logistical limitations and time constraints to including in-person interpretation in regular patient interactions. Nonetheless, both onsite and remote interpreters can help manage the flow of communication more easily for LEP patients. In fact, a systematic review of eight patient satisfaction studies showed that there is no significant difference in patient satisfaction between in-person interpreting and telephone interpreting [24]. Video interpreting, in particular, had the same level of patient satisfaction as in-person interpreting. Beyond face-to-face patient-provider interactions, interpretation services can be valuable for telehealth patient care. The COVID-19 pandemic in March 2020 drove telehealth expansion, congressional alterations to Medicare restrictions on telemedicine reimbursements, and platform access with subsequent state and private payor action,. These changes and long months of shelter in place and recurrent surges of COVID-19 infections caused a boom in telemedicine encounters [25]. Despite this rise in virtual visits, LEP patients continue experiencing challenges accessing telemedicine [26]. Telemedicine will likely remain prevalent, and healthcare professionals must be able to effectively incorporate interpreters into these patient encounters. A 2023 study developed an online module with didactic information and video examples as part of a longitudinal health equity curriculum for third-year medical students, with a post-module survey revealing student appreciation of the module’s efficacy [27]. This study can be used as a basic framework to integrate synchronous curricular experiences to teach students how to successfully work with interpreters during a virtual visit. Further investigation could also focus on assessing the long-term impact of incorporating real LEP patients and medical interpreters in pre-clerkship curricula on student learning outcomes through clinical rotations and even residency. Longitudinal studies with larger sample sizes could help determine if medical and PA students feel more confident working with interpreters and LEP patients in later clinical settings because of early training. Surveys could also be administered to the LEP patients themselves to gauge their satisfaction and comfort communicating with healthcare providers through interpreters.

There are limitations and opportunities for improvement in this study. First, the surveys only evaluated retrospective and self-rated levels of student confidence when working with interpreters. Future studies could incorporate the patients’ or the providers’ perceptions of the students’ ability to interact with LEP patients and interpreters. Moreover, a self-reported pre- and post- practicum assessment could be administered and analyzed to determine the change in students’ perceptions of their adeptness in working with LEP patients. Finally, it would be useful to repeat the survey, make it mandatory for all participants in the clinical sessions, or provide an incentive to maximize the response rate. In this study, the modest response rate, especially for students, may have introduced some nonresponse bias or participant selection bias. As the survey was anonymous with identifying information behind a firewalled system, it is not feasible to publicly distribute demographic data that might help clarify characteristics (e.g. nativity, race, or ethnicity) of responders versus non-responders. However, it is worth noting that this very anonymity, as well as the optional nature of the survey, minimizes the likelihood of bias introduced by students feeling pressured to report positive experiences to any potential evaluators, potentially enhancing the authenticity of the gathered responses. Since the survey was conducted at one medical school, our conclusions also may not be generalized to all medical schools. For improvements of the clinical practicum sessions themselves, many preceptors and interpreters mentioned the time constraint involved when incorporating medical interpreters into the clinical sessions; interpreters specifically wished to have the opportunity to introduce their role and background information to the student prior to the patient interview. More practice can also be done to ensure that the flow of the encounter is optimized. Some students mentioned that they would appreciate more sessions to be able to practice working with interpreters in different scenarios in which different modes of interpretation (simultaneous, consecutive, etc.) are employed [28].

It is also crucial to acknowledge the challenges that may preclude expanded implementation of this study’s curricular program by other medical schools. The administrative burden is initially high to set up the program, as this intervention requires active collaboration with an interpreter services team. Interpreter services may, as is the case in this program, require financial compensation. However, once established, the program’s maintenance is low and working with interpreters and LEP patients can be run simultaneously within clinical sessions, making it feasible to incorporate into most medical curricula. Some hospitals or health centers may not have a sufficient population of LEP patients, such as Veteran Affairs (VA) clinics.

Finally, there is increasing language equity research in healthcare that describes the limitation of reliance on English proficiency as a standard and categorizing individuals with the term “LEP” [29]. This label places the burden on the patient, potentially overlooking those with varying communication proficiencies or those who would benefit from non-English language. Some researchers encourage the replacing the term English-proficient or LEP with a more nuanced metric, such as non-English language preference, which focuses on language assets rather than deficits. While these are valid insights regarding the limitations of using the term “LEP” in healthcare research, there are practical reasons for its continued utilization in our study. Maintaining consistency with the terminology employed by our medical school program ensures coherence with our existing institutional frameworks, and the term currently remains a pragmatic and recognized descriptor in healthcare literature and policy for easier comparability with existing research. This facilitates the integration of our study findings into the broader body of literature, contributing to the ongoing discourse on language barriers in healthcare.

## Conclusion

Ultimately, this training with LEP patients and interpreter instruction provides students with communication skills that address the US population’s rapid growth in linguistic and cultural diversity. Medical schools and other professional curricula have an ethical obligation to adequately prepare the next generation of healthcare professionals to meet these linguistic demands and ensure equitable patient care.

### Supplementary Information

**Additional file 1.** This PDF file displays the survey administered to students.

**Additional file 2.** This PDF file displays the survey administered to preceptors.

**Additional file 3.** This PDF file displays the survey administered to interpreters.

## Data Availability

No datasets were generated or analysed during the current study.

## Abbreviations

CLAS: Culturally and Linguistically Appropriate Services
LCME: Liaison Committee on Medical Education
LEP: Limited-English Proficient
PA: Physician Assistant
POM: Practice of Medicine
SQUIRE: Standards for Quality Improvement Reporting Excellence
US: United States
