# Supplementary material for: Interpreter and limited-English proficiency patient training helps develop medical and physician assistant students’ cross-cultural communication skills
Source: BMC Med Educ. 2024 Feb 23;24:185. doi: 10.1186/s12909-024-05173-z (PMC10893691; doi:10.1186/s12909-024-05173-z)
Supplement: Supplementary file 1 — Additional file 1. This PDF file displays the survey administered to students. [file 12909_2024_5173_MOESM1_ESM.pdf]

Introduction

POM Y2 Practicum - Student Evaluation

Thank you for your willingness to reflect on your experience working with an interpreter and a limited English proficiency patient during clinical practicum this year. This survey is anonymous, and your answers will **not** be connected to your name or email address.

Student Evaluation

1. Please rank your agreement with the following statements on a scale from 1 (strongly disagree) to 5 (strongly agree).

	1	2	3	4	5
I understood what was expected of me during the POM Y2 practicum sessions during which I worked with an interpreter.	○	○	○	○	○
I incorporated the interpreter's recommendations into my interaction with the patient.	○	○	○	○	○
I am more confident working with an interpreter after this experience.	○	○	○	○	○
I benefitted from the					

feedback I received from the interpreter after interviewing the patient.	○	○	○	○	○
I would recommend that POM Y2 continue incorporating limited English proficiency patients and interpreters into clinical practicum.	○	○	○	○	○

2. What did you appreciate about this experience?

3. What techniques did you learn for working with an interpreter?

4. What was challenging about this experience?

5. How could this experience be improved for the students (e.g. changes to communication, resources you would recommend be provided as preparation for the encounter)?

6. What could the faculty do differently to improve the experience?

7. What could the interpreter do differently to improve the experience?

8. What other comments would you like to provide?

Powered by Qualtrics
