# Supplementary material for: Interpreter and limited-English proficiency patient training helps develop medical and physician assistant students’ cross-cultural communication skills
Source: BMC Med Educ. 2024 Feb 23;24:185. doi: 10.1186/s12909-024-05173-z (PMC10893691; doi:10.1186/s12909-024-05173-z)
Supplement: Supplementary file 2 — Additional file 2. This PDF file displays the survey administered to preceptors. [file 12909_2024_5173_MOESM2_ESM.pdf]

Introduction

POM Y2 Practicum - Preceptor Evaluation

Thank you for your willingness to reflect on your experience working with an interpreter and a limited English proficiency patient during clinical practicum this year. This survey is anonymous, and your answers will **not** be connected to your name or email address.

Faculty Evaluation

1. Please rank your agreement with the following statements on a scale from 1 (strongly disagree) to 5 (strongly agree).

	1	2	3	4	5
I understood what was expected of me during the POM Y2 practicum sessions during which we worked with an interpreter.	○	○	○	○	○
The interpreter provided recommendations prior to seeing the patient.	○	○	○	○	○
The students benefitted from the instruction provided by the interpreter prior to seeing the patient.	○	○	○	○	○

The students incorporated the recommendations for working with limited English proficiency patients and interpreters.	○	○	○	○	○
The students benefitted from the feedback the interpreter provided after interviewing the patient.	○	○	○	○	○
The inclusion of limited English proficiency patients and interpreters prepares medical students for similar interactions as practicing physicians.	○	○	○	○	○
I would recommend that POM Y2 continue incorporating limited English proficiency patients and interpreters into clinical practicum.	○	○	○	○	○

2. What did you appreciate about this experience?

3. What do medical students gain from the inclusion of limited English proficiency patients and interpreters in the POM Y2 clinical practicum?

4. What challenges did you encounter while guiding students through working with an interpreter and a limited English proficiency patient?

5. How could this experience be improved for the students?

6. How could this experience be improved for the faculty (e.g. changes to workflow/communication, resources you would recommend be provided as preparation for the encounter)?

7. What other comments would you like to provide?

Powered by Qualtrics
