# Supplementary material for: Interpreter and limited-English proficiency patient training helps develop medical and physician assistant students’ cross-cultural communication skills
Source: BMC Med Educ. 2024 Feb 23;24:185. doi: 10.1186/s12909-024-05173-z (PMC10893691; doi:10.1186/s12909-024-05173-z)
Supplement: Supplementary file 3 — Additional file 3. This PDF file displays the survey administered to interpreters. [file 12909_2024_5173_MOESM3_ESM.pdf]

Introduction

POM Y2 Practicum - Interpreter Evaluation

Thank you for your willingness to reflect on your experience working with an interpreter and a limited English proficiency patient during clinical practicum this year. This survey is anonymous, and your answers will **not** be connected to your name or email address.

Interpreter Evaluation

1. Please rank your agreement with the following statements on a scale from 1 (strongly disagree) to 5 (strongly agree).

	1	2	3	4	5
I understood what was expected of me during the POM Y2 practicum sessions.	○	○	○	○	○
The preceptor invited my input in the discussion before we interviewed the patient.	○	○	○	○	○
The students incorporated my recommendations when working with limited English proficiency patients and interpreters.	○	○	○	○	○

The students benefitted from the feedback I provided after interviewing the patient.	○	○	○	○	○
I would recommend that POM Y2 continue incorporating limited English proficiency patients and interpreters into clinical practicum.	○	○	○	○	○

2. What did you appreciate about this experience?

3. How could this experience be improved for the students?

4. How could this experience be improved for the interpreters (e.g. changes to workflow/communication, resources you would recommend be provided as preparation for the encounter)?

5. What could the faculty do differently to improve the experience?

6. What other comments would you like to provide?

Powered by Qualtrics
